# *EGFR* Mutation Testing in Patients with Advanced Non-Small Cell Lung Cancer: A Comprehensive Evaluation of Real-World Practice in an East Asian Tertiary Hospital

**DOI:** 10.1371/journal.pone.0056011

**Published:** 2013-02-28

**Authors:** Yoon-La Choi, Jong-Mu Sun, Juhee Cho, Sanjay Rampal, Joungho Han, Bhash Parasuraman, Eliseo Guallar, Genehee Lee, Jeeyun Lee, Young Mog Shim

**Affiliations:** 1 Department of Pathology, Samsung Medical Center, Sungkyunkwan University School of Medicine, Seoul, Republic of Korea; 2 Department of Health Sciences and Technology, Samsung Advanced Institute for Health Sciences and Technology, Sungkyunkwan University, Seoul, Republic of Korea; 3 Division of Hematology-Oncology, Department of Medicine, Samsung Medical Center, Sungkyunkwan University School of Medicine, Seoul, Republic of Korea; 4 Department of Health, Behavior and Society, Johns Hopkins Bloomberg School of Public Health, Baltimore, Maryland, United States of America; 5 Department of Epidemiology, Johns Hopkins Bloomberg School of Public Health, Baltimore, Maryland, United States of America; 6 Department of Medicine, and Welch Center for Prevention, Epidemiology, and Clinical Research, Johns Hopkins Bloomberg School of Public Health, Baltimore, Maryland, United States of America; 7 Julius Centre University of Malaya, Department of Social and Preventive Medicine, Faculty of Medicine, University of Malaya, Kuala Lumpur, Malaysia; 8 AstraZeneca, Wilmington, Delaware, United States of America; 9 Department of Nursing, Samsung Medical Center, Sungkyunkwan University School of Medicine, Seoul, Republic of Korea; 10 Department of Thoracic Surgery, Samsung Medical Center, Sungkyunkwan University School of Medicine, Seoul, Republic of Korea; Sanjay Gandhi Medical Institute, India

## Abstract

**Introduction:**

Guidelines for management of non-small cell lung cancer (NSCLC) strongly recommend *EGFR* mutation testing. These recommendations are particularly relevant in Asians that have higher *EGFR* mutation prevalence. This study aims to explore current testing practices, logistics of testing, types of *EGFR* mutation, and prevalence of *EGFR* mutations in patients with advanced NSCLC in a large comprehensive cancer center in Korea.

**Methods:**

Our retrospective cohort included 1,503 NSCLC patients aged ≥18 years, with stage IIIB/IV disease, who attended the Samsung Medical Center in Seoul, Korea, from January 2007 through July 2010. Trained oncology nurses reviewed and abstracted data from electronic medical records.

**Results:**

This cohort had a mean age (SD) of 59.6 (11.1) years, 62.7% were males, and 52.9% never-smokers. The most common NSCLC histological types were adenocarcinoma (70.5%) and squamous cell carcinoma (18.0%). Overall, 39.5% of patients were tested for *EGFR* mutations. The proportion of patients undergoing EGFR testing during January 2007 through July 2008, August 2008 through September 2009, and October 2009 through July 2010 were 23.3%, 38.3%, and 63.5%, respectively (P<0.001). The median time elapsed between cancer diagnoses and receiving *EGFR* testing results was 21 days. *EGFR* testing was most frequently ordered by oncologists (57.7%), pulmonologists (31.9%), and thoracic surgeons (6.6%). *EGFR* testing was more commonly requested for women, younger patients, stage IV disease, non-smokers, and adenocarcinoma histology. Of 586 cases successfully tested for *EGFR* mutations, 209 (35.7%) were positive, including 118 cases with exon 19 deletions and 62 with L858R mutations. *EGFR* mutation positive patients were more likely to be female, never-smokers, never-drinkers and to have adenocarcinoma.

**Conclusions:**

In a large cancer center in Korea, the proportion of *EGFR* testing increased from 2007 through 2010. The high frequency of *EGFR* mutation positive cases warrants the need for generalized testing in Asian NSCLC patients.

## Introduction

In non-small-cell lung cancer (NSCLC), the increased responsiveness of epidermal growth factor receptor (EGFR) mutation positive cases to EGFR-tyrosine kinase inhibitors, such as gefitinib or erlotinib, represents a landmark finding towards personalized lung cancer care [1]–[6]. Indeed, since early 2011, the American Society of Clinical Oncology (ASCO) and the National Comprehensive Cancer Network (NCCN) recommend *EGFR* mutation testing for patients with advanced non-squamous NSCLC [7], [8]. *EGFR* mutation positive NSCLC cases are more common in Asian compared to Western patients, heightening the need for testing all NSCLC patients.

In routine clinical practice, however, *EGFR* mutation testing requires substantial resources and effort, including availability of qualified pathologists and molecular biologists, effective clinical coordination, physician and patient time, and associated costs. In addition, tissue samples may be unavailable or insufficient for testing [9], and there may be additional organizational barriers to widespread implementation of *EGFR* testing. Although *EGFR* testing is becoming more common, there have been few systematic assessments of performance practices of *EGFR* mutation testing in real-world clinical settings. The objective of this study was to explore current testing practices and prevalence of *EGFR* in patients with advanced NSCLC in a large comprehensive cancer center in Korea, and to describe the types and logistics for *EGFR* mutation testing, including where the tests were conducted and the delay between requesting and reporting test results.

## Methods

### Study Design and Patients

We conducted a retrospective cohort study of all stage IIIB and IV NSCLC patients with confirmed histologic or pathologic diagnosis admitted to the Samsung Medical Center (Seoul, Korea) from January 2007 through July 2010 for the diagnosis or treatment of NSCLC (N = 1,503). The study was approved by the Institutional Review Board of the Samsung Medical Center. The requirement of informed consent was waived, as the study was based on existing administrative and clinical data.

### Data Collection

Study data were abstracted, using in-hospital charts and electronic medical records, by trained experienced nurses from the Departments of Medical Oncology, Surgical Oncology, Laboratory Medicine, Pathology, and Nursing at the Samsung Medical Center. Baseline clinical characteristics included gender, age at diagnosis, smoking history, alcohol consumption status, date of diagnosis of advanced lung cancer, tumor histology, tumor stage, and ambulatory status at diagnostic work-up. Tumor stage was defined according to the sixth edition of the American Joint Committee on Cancer (AJCC). Regarding performance practices for *EGFR* mutation test, we obtained the request history and date, report date, and test results including type of *EGFR* mutation.

Smoking history and alcohol consumption status were based on self-reported questionnaires. Never smokers were defined as patients who smoked <100 cigarettes over their life-time. Former smokers were patients who had smoked ≥100 cigarettes in their lifetime but had stopped smoking for at least 1 year before the diagnosis of lung cancer. Never drinkers were patients who had never consumed any alcohol in their lifetime.

### *EGFR* Mutation Testing

*EGFR* mutation testing was performed by ISU ABXIS Co. (Seoul, Korea), an independent commercial laboratory, before August 2008, and by the Department of Pathology at the Samsung Medical Center thereafter. The mutational analyses of *EGFR* (exons 18–21) were performed by directional sequencing of polymerase chain reaction (PCR) fragments amplified with genomic DNA from paraffin-embedded tissue. PCR was performed in a 20 µL volume containing 100 ng of template DNA, 10 x PCR buffer; 0.25 mM dNTPs, 10 pmol primers and 1.25 U Taq DNA polymerase (iNtRON, Korea). PCR products were electrophoresed on 2% agarose gels and were purified with a QIAquick PCR purification kit (QIAGEN, Hilden, Germany). Bidirectional sequencing was performed using the BigDye Terminator v 1.1 kit (Applied Biosystems, Foster City, CA, USA) on an ABI 3130xl genetic analyzer (Applied Biosystems, Foster City, CA, USA).

### Statistical Analyses

We used means and standard deviations (SD) to summarize symmetrically distributed variables, medians and interquartile ranges to summarize skewed variables, and counts and percentages to summarize categorical variables. Differences in proportions and medians were statistically tested using χ^2^ square and Kruskal Wallis tests, respectively. The determinants of requesting *EGFR* mutation testing in the overall patient population, and the determinants of a positive test among patients who were tested, were evaluated using Poisson regression with robust standard errors. The prevalence ratios for requesting EGFR mutation testing were adjusted for gender, age at diagnosis (categorized as <65 and ≥65 years), smoking status (categorized as current, former, and never smokers), mobility at admission (categorized as wheelchair/bed and ambulatory), disease stage (categorized as stage IIIB and IV), tumor histology, (categorized as squamous cell carcinoma, adenocarcinoma, and other histology) and period of admission (categorized as 2007/01–2008/07, 2008/08–2009/09, and 2009/10–2010/07). The prevalence ratios for positive EGFR mutation status were adjusted for gender, age at diagnosis, smoking status, and tumor histology. Statistical analyses were performed using Stata statistical software version 12. Two-sided p values<0.05 were considered statistically significant.

## Results

### Patient Population

The mean age (SD) of the 1,503 patients was 59.6 (11.1) years, 62.7% were males and most were of Korean ethnicity (99.3%) (Table 1). At the time of admission for diagnostic work-up, 36.6% of patients were current smokers and 81.1% were able to walk in. Most patients (70.9%) had stage IV NSCLC with 651 (43.3%) patients having metastasis to multiple sites. Common metastatic sites were bone (43.3%), lung (39.1%), brain (25.1%), and liver (10.8%). The most common histology was adenocarcinoma (70.5%), followed by squamous cell carcinoma (18.0%). Diagnostic methods included chest x-ray (100%), chest computed tomography (100%), PET (82.0%), MRI (81.0%), bronchoscopy (62.5%), and bone scan (20.2%).

**Table 1:** Baseline characteristics of study patients.

Characteristics	Number (%) or mean (SD)				
	All patients	*EGFR* mutation positive	*EGFR* mutation negative	*EGFR* mutation unknown	P
	N = 1,503	N = 209	N = 366	N = 928	
Mean age, years	59.6 (11.1)	56.5 (11.0))	58.5 (11.1)	60.7 (10.9)	<0.001
Gender					<0.001
Male	943 (62.7)	87 (41.6)	214 (58.5)	642 (69.2)	
Female	560 (37.3)	122 (58.4)	152 (41.5)	286 (30.8)	
Ethnicity					0.87
Korean	1492 (99.3)	208 (99.5)	362 (98.9)	922 (99.4)	
Caucasian	9 (0.6)	1 (0.5)	3 (0.8)	5 (0.5)	
Other Asians	2 (0.1)	0 (0.0)	1 (0.3)	1 (0.1)	
Smoking history					<0.001
Never	697 (52.9)	131 (69.3)	181 (53.4)	385 (48.7)	
Former (Stopped for >1 year)	138 (10.5)	21 (11.1)	45 (13.3)	72 (9.1)	
Current	483 (36.6)	37 (19.6)	113 (33.3)	333 (42.2)	
Alcohol consumption					0.019
Never	787 (59.8)	130 (68.8)	186 (54.9	471 (59.7)	
Former (Stopped for ≥3 months)	75 (5.7)	5 (2.6)	20 (5.9)	50 (6.3)	
Current	455 (34.5)	54 (28.6)	133 (39.2)	268 (34.0)	
Stage					0.003
IIIB	438 (29.1)	41 (19.6)	104 (28.4)	293 (31.6)	
IV	1065 (70.9)	168 (80.4)	262 (71.6)	635 (68.4)	
Tumor histology					<0.001
Adenocarcinoma	1059 (70.5)	200 (95.7)	287 (78.4)	572 (61.6)	
Squamous cell carcinoma	270 (18.0)	6 (2.9)	48 (13.1)	216 (23.3)	
Others	174 (11.6)	3 (1.4)	31 (8.5)	140 (15.1)	
Mobility at admission for initial diagnosis					0.02
Ambulatory	1071 (81.1)	164 (86.8)	280 (82.1)	627 (79.3)	
On a wheelchair	114 (8.6)	16 (8.5)	32 (9.4)	66 (8.3)	
On a bed	136 (10.3)	9 (4.8)	29 (8.5)	98 (12.4)	

The number (%) of patients with missing data were 185 (12.3%) for smoking, 186 (12.4%) for alcohol consumption, and 182 (12.1%) for mobility at admission for initial diagnosis.

### Performance of *EGFR* Mutation Testing

A total of 593 (39.5%) patients were referred for *EGFR* mutation test. Mutations in exons 18 to 21 could not be tested in seven cases, resulting in 586 patients (39.0%) successfully tested. An additional 11 patients, not tested for both exon 19 and 21 mutations, were classified as *EGFR* mutation-unknown. This is because two typical activating mutations (in-frame deletion and L858R mutation) are located in exons 19 and 21, respectively. *EGFR* mutation status was available for 575 (38.3%) patients. The median time elapsed between histologic diagnosis of NSCLC and ordering *EGFR* mutation testing was 21 days (interquartile range [IQR], 12–56 days).

Women, former smokers, those with better mobility when admitted for diagnostic work-up, and those with stage IV disease or adenocarcinoma histology were significantly more likely to have *EGFR* mutation testing requested (Table 2). To evaluate temporal trends in *EGFR* mutation testing, we divided the study in 3 periods: from January 2007 through July 2008 (*EGFR* testing was requested to an outside laboratory during this period), from August 2008 through September 2009 (publication of the IPASS trial [2]), and from October 2009 through July 2010. The proportions of patients referred for *EGFR* mutation testing in each of these periods were 23.3%, 38.3%, and 63.5%, respectively (P<0.001). *EGFR* testing was most commonly ordered by oncologists (57.7%) and pulmonologists (31.9%), followed by thoracic surgeons (6.6%).

**Table 2:** Prevalence ratios (95% confidence intervals) for request of *EGFR* mutation testing by patient characteristics.

Characteristics	N	No. of request (%)	Crude Prevalence Ratio (95%CI)	P	Adjusted Prevalence Ratio* (95% CI)	P
Gender						
Male	943	311 (33.0)	1.00		1.00	
Female	560	282 (50.4)	1.53 (1.35–1.73)	<0.001	1.32 (1.11–1.56)	0.001
Age at Diagnosis						
≥65 yrs	542	175 (32.3)	1.00		1.00	
<65 yrs	961	418 (43.5)	1.35 (1.17–1.55)	<0.001	1.13 (0.98–1.29)	0.09
Smoking Status						
Current smoker	483	156 (32.3)	1.00		1.00	
Former smoker	138	68 (49.3)	1.53 (1.23–1.89)	<0.001	1.22 (1.01–1.48)	0.04
Never smoker	697	322 (46.2)	1.43 (1.23–1.67)	<0.001	1.08 (0.89–1.29)	0.45
Mobility at admission for initial diagnosis						
Wheelchair/bed	250	89 (35.6)	1.00		1.00	
Ambulatory	1071	459 (42.9)	1.20 (1.00–1.44)	0.04	1.24 (1.05–1.47)	0.013
Stage						
IIIB	438	149 (34.0)	1.00		1.00	
IV	1065	444 (41.7)	1.23 (1.06–1.42)	0.007	1.25 (1.09–1.44)	0.002
Histology						
Squamous cell carcinoma	270	57 (21.1)	1.00		1.00	
Adenocarcinoma	1059	502 (47.4)	2.25 (1.77–2.85)	<0.001	1.90 (1.50–2.41)	<0.001
Other Histology	174	34 (19.5)	0.93 (0.63–1.35)	0.69	0.92 (0.63–1.34)	0.67
Period of admission						
2007/01–2008/07	562	131 (23.3)	1.00		1.00	
2008/08–2009/09	538	206 (38.3)	1.64 (1.37–1.98)	<0.001	1.61 (1.34–1.93)	<0.001
2009/10–2010/07	403	256 (63.5)	2.73 (2.31–3.22)	<0.001	2.57 (2.16–3.06)	<0.001

* Multivariate analysis included 1,315 patients, and adjusted for gender, age at diagnosis, smoking status, mobility at admission, disease stage, tumor histology, and period of admission.

Among patients referred for *EGFR* mutation testing prior to August 2008, between August 2008 and September 2009, and after September 2009, the median times (IQR) from diagnosis to *EGFR* testing request were 31 (16–374), 23 (14–117), and 16 days (9–29.5), respectively (P<0.001) and the median times from lung cancer diagnosis to *EGFR* mutation report were 49 (30–416), 35 (23–131), and 32 days (23–49), respectively (P<0.001). Among adenocarcinomas, the prevalence of *EGFR* positive tumors in each period was 45.5% (N = 51), 38.2% (N = 66) and 41.1% (N = 83), respectively (P = 0.47).

### *EGFR* Mutation Types

Among 575 patients with available *EGFR* mutation status, 209 (36.3%) were mutation positive (Table 3). Fourteen patients had double mutations, simultaneously or subsequently, in the same (N = 5) or in different exons (N = 9). All mutations in exon 18 (N = 15) were of the missense type, mostly composed of G719A (S or C) and S720F (P). Among mutations in exon 19 (N = 123), 120 were typical in-frame deletions around c.2230–2250, and two and one were insertion and missense types, respectively. Among mutations in exon 20 (N = 18), 8 were of the missense type, 4 were of the insertion type, 3 were of the duplication type, and two were double mutations. All mutations in exon 21 (N = 62) were of the missense types (61 L858R and one L861Q).

**Table 3:** *EGFR* mutation types and their distribution.

Distribution of EGFR mutations	Number of patients (%)
Patients successfully tested	586
Patients with available data	575
Negative *EGFR* mutation	366
Unknown *EGFR* mutation†	11
Positive EGFR mutation*	209/575 (36.3%)
Tumor histology	
In Adenocarcinoma	203/521(39.0%)
In Squamous cell carcinoma	6/54 (11.1%)
Exons	
Exon 18	15/575 (2.6%)
G719A	6
G719A and S720F (double)	1
G719S and A721T (double)	1
G719S and E709A (double)	1
G719C	1
S720F	2
S720P	2
F723L	1
Exon 19	123/580 (21.2%)
Deletion	120
Ins TTAAAATTCCCATCGCTG (c.2231–2248)	1
Ins AAAATTCCCGTCGCTATC (c.2232–2233)	1
I744M	1
Exon 20	18/574 (3.1%)
T790M	3
S719I	1
S768I and V774M (double)	1
V786M	1
A776H	1
R776S	1
G796S	1
Ins ACC (c.2314–2316)	1
Ins GTT (c.2309–2311) and P772H (double)	1
Ins AACTCC (c.2317–2322)	1
Ins GCCAGCGTG (c.2308–2309)	1
Ins GGC (c.2313–2314)	1
Del Ins TTCCAGGAAGTC TACGTGATGGA (c. 2291–2300)	1
Dup (nt. 2310–23110)	1
Dup CCAGCGTG (c.2300–2308)	1
Dup CCAGCGTGC (c.2209–2317)	1
Exon 21	62/573 (10.8%)
L858R	61
L861G	1

* Fourteen patients had double mutation types simultaneously or sequentially.

† Patients were classified as *EGFR* mutation-unknown if they were not tested for both exon 19 and exon 21 mutations.

### Relationship between *EGFR* Mutation Status and Clinical Characteristics

Patients with adenocarcinoma (41.1%) had significantly higher proportions of positive *EGFR* mutation tests compared to those with squamous cell carcinoma (11.1%) (P<0.001). In crude analyses, women, patients who were younger, never smokers, and those with adenocarcinoma histology were significantly more likely to test positive for *EGFR* mutations (Table 4). After adjustment, however, only adenocarcinoma histology was significantly associated with positive *EGFR* mutation (adjusted prevalence ratio = 2.91; 95% CI 1.34–6.32; P = 0.007).

**Table 4:** Prevalence ratios (95% confidence intervals) for positive *EGFR* mutation status among 575 patients with available *EGFR* mutation data.

Covariates	N	No. of positive EGFR mutation n, (%)	Crude Prevalence Ratio (95% CI)	P	Adjusted Prevalence Ratio*(95% CI)	P
Gender						
Male	301	87 (28.9%)	1.00		1.00	
Female	274	122 (44.5%)	1.54 (1.23–1.92)	<0.001	1.20 (0.87–1.64)	0.26
Age at diagnosis						
≥65 yrs	169	50 (29.6%)	1.00		1.00	
<65 yrs	406	159 (39.2%)	1.32 (1.02–1.72)	0.036	1.25 (0.95–1.64)	0.11
Smoking status						
Current smoker	150	37 (24.7%)	1.00		1.00	
Former smoker	66	21 (31.8%)	1.29 (0.82–2.02)	0.27	1.36 (0.89–2.07)	0.16
Never smoker	312	131 (42.0%)	1.70 (1.25–2.32)	0.001	1.31 (0.89–1.93)	0.17
Histology						
Squamous cell carcinoma	54	6 (11.1%)	1.00		1.00	
Adenocarcinoma	487	200 (41.1%)	3.70 (1.72–7.92)	0.001	2.91 (1.34–6.32)	0.007
Other histology	34	3 (8.8%)	0.79 (0.21–2.97)	0.73	0.51 (0.11–2.35)	0.39

* Multivariate analysis included 528 patients, and adjusted for gender, age at diagnosis, smoking status, and histology.

Among 54 patients with squamous cell carcinoma and available *EGFR* mutation data, 43 patients were male, 14 were never smokers, and 6 tested positive for *EGFR* mutations (one double mutation [G719A and S720P] and F723L in exon 18; four typical in-frame deletions in exon 19; and one V786M mutation in exon 20). Three of the six patients with *EGFR* mutation positive squamous cell carcinoma were never smokers and all of them had typical in-frame deletions in exon 19. The proportion of positive *EGFR* mutations among never-smokers with squamous cell carcinoma was 16.7% (2/12),

## Discussion

In this large real-world cohort of advanced stage NSCLC from a tertiary hospital in Korea, 39.5% of patients were tested for *EGFR* mutation. The frequency of testing increased over time, such that between October 2009 and July 2010 the proportion of patients referred for testing was 63.5%. Since most histologic diagnoses were based on a small amount of specimen obtained from bronchoscopy, needle biopsy, and aspiration, the testing rate during this period can be considered high. Indeed, Lynch et al. concluded in 2010 that *EGFR* mutation testing was vastly underused in American NSCLC patients [10].

In our center, *EGFR* mutation testing was requested more frequently and more promptly over the years. Two factors may have influenced these time trends. First, our center developed the logistic capabilities for performing both histology and *EGFR* testing in the same pathology laboratory, thus reducing request complexity and turn-around times. Second, in agreement with the publication of landmark studies and national practice guidelines, different members in multidisciplinary NSCLC care teams at our center, including medical oncologists, pulmonologists, thoracic surgeons, and pathologists, agreed to the importance of improving the clinical performance of *EGFR* mutation testing.

We also found that request rates for *EGFR* mutation testing were associated with a number clinical characteristics of study patients. Test requests were more likely in patients with adenocarcinoma, and in those who were younger, female, never/former smokers, or who were ambulatory on admission. The association between request rates with age or performance status was likely due to concerns on side effects and complications associated with more aggressive biopsy procedures required to obtain specimens for *EGFR* mutation testing in patients with poorer performance status.

The NCCN guidelines do not recommend *EGFR* mutation testing in squamous cell carcinoma [8]. A previous study conducted in Italy found no *EGFR* mutations among 454 patients with squamous cell carcinoma [11]. In contrast, our study found that 6 out of 54 patients (11.1%) with squamous cell carcinoma had *EGFR* mutations, with an even higher prevalence in the subgroup of never smoker patients with squamous cell carcinoma (2 out of 12, or 16.7%). Recently, Tanaka et al. [12] reported a case in which a male current smoker with an EGFR mutation positive squamous cell carcinoma and performance status 4 showed a marked response to first-line gefitinib therapy. Our findings suggest that *EGFR* mutation testing should be considered even for squamous cell carcinoma patients in East Asian populations, although further studies need to better characterize the prevalence of *EGFR* mutation testing in East Asian patients with squamous cell lung carcinoma.

Although our data showed that *EGFR* mutation testing was quickly becoming a standard part of routine management in NSCLC patients in our center during 2010, several barriers to improve quality of care remained. First, additional coordination and logistic efforts are needed to decrease turn-around times from diagnosis of advanced NSCLC to *EGFR* mutation test reporting in order to maximize its clinical utility. Second, even though *EGFR* mutation testing provides key information for decision making in treating NSCLC patients, testing is not reimbursed in Korea, a country in which health care is largely covered by a single-payer public insurance system although provision of care is provided in private centers. With an approximate cost of US $200, the economic burden may discourage a number of NSCLC patients from getting *EGFR* testing. Reimbursement of *EGFR* testing by insurance is important to further extend testing, particularly since more active molecular analyses, which search for additional predictive biomarkers such as EML4-ALK translocations [13], [14], are becoming clinically available.

Our study was limited to a single tertiary hospital in Seoul, with a large volume of inpatient and outpatient consultations. Our findings may not be generalizable to countries outside of East Asia or to lower volume centers. However the key findings in our study, including the prevalence of *EGFR* mutations and the clinical characteristics of mutation positive patients, are expected to be similar to other major East Asian medical facilities. The retrospective nature of our study is also a potential limitation. Several strengths, however, including the large number of patients, the evaluation of consecutive patients in a real-world practice setting, and the availability and extraction of detailed clinical notes regarding testing procedures, add to the relevance of our findings.

Recently, Sun et al. [15] reported that the frequency of the EGFR gene mutation is quite high among Korean patients with adenocarcinoma (up to 68.5% in nonsmoker women with adenocarcinoma) and even in male smokers with adenocarcinoma (29.7%). The current consensus recommends testing all newly diagnosed patients with advanced stage non squamous lung cancer, as well as some patients with squamous cell carcinoma with clinical features associated with higher prevalence of EGFR mutations in East Asia [9].

Finally, there are several issues in performance of EGFR mutation testing in practical environments, including sample type, techniques, turnaround time, and cost [16]–[18]. Regardless of these issues, Gately et al. showed that testing performed in the same center where the patient has been pathologically diagnosed was associated with shorter testing lead times and lower probability of result misallocation [19].

In conclusion, in this comprehensive analysis of real-world practice regarding *EGFR* mutation testing in East Asia, we found that 39.5% of advanced NSCLC patients attending our center were tested for *EGFR* mutations from January 2007 to July 2010. The frequency of *EGFR* mutation testing increased over time, and by 2010 it had become part of the clinical workup in the majority of NSCLC patients. In accordance with the increase in the frequency of testing over time, the turn-around time from cancer diagnosis to *EGF*R mutation report progressively decreased, facilitating the clinical use of test results. Finally, while the prevalence of *EGFR* mutation positivity was 39% among adenocarcinoma patients, we found a that 11% of patients with squamous cell carcinoma also tested positive, suggesting that testing should be generalized to all NSCLC patients in East Asia.
